# Dynamic Dazzle Distorts Speed Perception

**DOI:** 10.1371/journal.pone.0155162

**Published:** 2016-05-19

**Authors:** Joanna R. Hall, Innes C. Cuthill, Roland Baddeley, Angela S. Attwood, Marcus R. Munafò, Nicholas E. Scott-Samuel

**Affiliations:** 1 School of Experimental Psychology, University of Bristol, Bristol, United Kingdom; 2 School of Biological Sciences, University of Bristol, Bristol, United Kingdom; University of Sussex, UNITED KINGDOM

## Abstract

Static high contrast (‘dazzle’) patterns, such as zigzags, have been shown to reduce the perceived speed of an object. It has not escaped our notice that this effect has possible military applications and here we report a series of experiments on humans, designed to establish whether dynamic dazzle patterns can cause distortions of perceived speed sufficient to provide effective defence in the field, and the extent to which these effects are robust to a battery of manipulations. Dynamic stripe patterns moving in the same direction as the target are found to increase the perceived speed of that target, whilst dynamic stripes moving in the opposite direction to the target reduce the perceived speed. We establish the optimum position for such dazzle patches; confirm that reduced contrast and the addition of colour do not affect the performance of the dynamic dazzle, and finally, using the CO_2_ challenge, show that the effect is robust to stressful conditions.

## Introduction

Camouflage is generally considered to be a mechanism for hiding an object ‘in plain sight’ [1,2]. However, there are other properties of objects that can also be hidden or distorted in order to gain protection, especially when the object is in motion [3–5]. For example, complex high contrast patterns were used on ships during both World Wars with the aim of disguising properties such as the direction, size, shape, range and speed [6,7] of the moving target. This so-called dazzle colouration was often a combination of different patterns including stripes (vertical, horizontal and diagonal), checks, zigzags and some less regular shapes. There was little contemporary empirical evidence for the success, or otherwise, of this dazzle strategy (although see [8]), but evidence for perceptual distortions created by single regular patterns suggests the possibility that at least some elements of the dazzle patterns could have proved successful [4,5,9–11]. Although navies are generally no longer reliant on the human visual system to detect and target other vessels, there are other scenarios where a dazzle strategy may still provide a useful benefit. For instance, moving vehicles are vulnerable to handheld weapons fired from short ranges. Here, disguising speed could make the difference between a near miss and a direct hit. We report a series of experiments designed to establish whether a dynamic texture can cause distortions of perceived speed sufficient to provide effective defence in the field, and the extent to which these effects are robust to a battery of manipulations.

In computer game experiments with human participants, stripes have been shown to significantly lower capture rates of moving targets, compared to some other patterns [4,5]. Scott-Samuel *et al*. [9] investigated whether dazzle patterns, such as stripes, zigzags and checks, were specifically able to distort the perceived speed of a target. Their results showed that at slow speeds, the patterns had no effect. However, when the target moved at higher speed (20 deg/s) there was evidence that some patterns could decrease the perceived speed by around 7%. This effect was reliant upon the pattern being both high contrast and sufficiently complex (zigzags and checks in their experiments). The size and speed of their target corresponded approximately to a Land Rover at 70m, travelling at 55mph. In this scenario, a 7% decrease in perceived speed would correspond to an approximate targeting error of 1m.

It is well established that a drifting pattern can induce illusory positional shifts of the region it covers, in the direction of the pattern motion [12–15]. The stimulus typically used for these experiments is a Gabor patch (a sinusoidal modulation of luminance–a luminance grating–within a two-dimensional Gaussian envelope) with a drifting carrier [12, 14, 15]. The effect is greatest when the pattern moves at high speed, but the size of the illusory positional shift is not directly proportional to the speed of the pattern [12].

Zhang *et al*. [16] looked at the effects of a moving luminance grating within a patch that was either stationary or moving. They reported that the moving grating induced illusory movement of the stationary patch, but the direction of this movement was dependent on the sharpness of boundary around the patch. When the patch was also moving, the motion of the grating was found to strongly affect the perceived strength of the patch's motion. While the direction of this effect was also highly dependent on the patch boundary sharpness, the velocity of the pattern, relative to the patch, has been reported as the primary determinant of the strength of the illusory shift [17].

These previous studies provide good evidence that a moving pattern within a moving object can produce illusory effects on either position or speed. However, in order for such a strategy to be applied in real world scenarios, the effect would need to be predictable, easily manipulated, reliable and also robust to the stressful conditions typically experienced during low-tech warfare. We combined the paradigms of Scott-Samuel *et al*. [9] and Zhang *et al*. [16] to create moving rectangular targets that contained a dynamic texture similar to a dazzle pattern. This dynamic texture could move in the same direction as the motion of the target, predicted to increase perceived speed, or it could move in the opposite direction to the motion of the target, predicted to decrease perceived speed.

In this series of experiments we used different manipulations to investigate the conditions under which the change in perceived target speed due to a dynamic texture is maximised. We investigated how the speed of the pattern affects the perceived speed of the target and the generalizability of these effects; whether patches of dynamic texture can also affect perceived speed and what the optimum position is for such patches; whether reduced contrast and the addition of colour affect the performance of dynamic texture and finally whether the strategy is robust to judgements made under stressful conditions.

## General Methods

In all experiments volunteers gave their informed written consent in accordance with the Declaration of Helsinki, and the experiments were approved by the Research Ethics Committee of the Faculty of Science, University of Bristol. All participants were naïve to the hypotheses being tested. Data have been deposited in the University of Bristol Research Data Repository, doi:10.5523/bris.1b4oomlq4xax315vub1set1kjj.

The stimulus generation and experimental control programs were written in Matlab (The Mathworks Inc., Natick, MA), using the Psychophysics Toolbox extensions [18, 19] on a MacBook Pro (Apple Inc.). Stimuli were viewed binocularly without a fixation point, and were displayed 62 cm from the subject on a linearized Iiyama Vision Master Pro 513 CRT monitor with a mean luminance of 65.6 cd/m^2^, a resolution of 1024 x 768 pixels (visual angle of 33 x 25 degrees) and a refresh rate of 100 Hz.

On each trial, subjects were presented with a two temporal interval, binary choice task, and reported (via a keypad) in which interval the stimulus moved more quickly. The speed of the test stimulus was constant, whilst that of the comparison stimulus was varied from trial to trial by the APE algorithm [20] in order to home in on the point of subjective equality. The comparison stimulus was a one-dimensional horizontal Gaussian luminance profile; this was used to allow for fine adjustment of its speed. Exclusion criteria were set prior to testing so that any participant producing a standard deviation of more than 2 pixels/frame (6.66 deg/sec) on any block would be excluded from the experiment. Excluded participants often produced particularly high or low perceived speeds and these could have had a large impact on the mean perceived speed as well as increasing the variation in the data. After the first experiment we compared the standard deviations of the mean change in perceived speed for the dataset with participants excluded and the dataset with all participants included. Inclusion of all the participants increased the standard deviations of the eight blocks by 55–97%. Each experiment was continued until fifteen admissible sets of data had been recorded. Participants either gained course credit or were reimbursed for their time.

As in Scott-Samuel *et al*. [9], the test stimuli were rectangles with dimensions 1.3 x 3.3 deg. Under our viewing conditions, this corresponds approximately to the size of a Land Rover viewed from a distance of 70m, a typical distance between a rocket propelled grenade launcher and its target [21].

Each of the test targets was wholly or partially covered by a patch of putative dazzle camouflage. This could be a fixed texture on the target, or move across its surface. Throughout we use the term target speed to refer to the movement of the target relative to the background (or, equivalently, the reference frame of the monitor’s edges), whereas we use the term texture speed to refer to the speed of the pattern on the target’s surface relative to the target’s edges. One could, in principle, think of the speed of the texture as relative to the background. This would be the simple sum of the texture speed, as defined by us, plus the target speed. However, because a texture is a property of an object, we feel it is more natural to refer to texture speed as relative to the moving target, not the background. The exact pattern, size, speed and direction of the movement of the texture depended on the particulars of the experiment. Only one test target type was used within any given block, and each block contained 64 trials. The order in which the blocks were shown was randomised. All stimuli were displayed on a mean luminance background (except in Experiment 6) and translated horizontally across the screen from a pseudo-random starting point, and for a randomly assigned duration (within the range 400–600 ms), in order to avoid distance travelled being used as a cue to stimulus speed. All randomisations were achieved using the Matlab ‘shuffle’ function to re-order a list of all the blocks to be tested.

## Experiment 1: Speed of Dynamic Camouflage

### Methods

In the first experiment the speed and direction of the dynamic pattern, relative to the target, were systematically varied. The texture was a vertically oriented, 100% contrast, sinusoidal modulation of luminance with a spatial frequency of 1.5 c/deg. It completely covered the target rectangle. The target itself moved at 10 deg/s.

In order to investigate a large range of texture speeds we ran two experiments. In each of these experiments there were nine blocks, one with a control dazzle texture that was fixed on the target’s surface, and eight with differing relative speeds and directions of dazzle texture. In Experiment 1a we tested four speeds of lower magnitude (0.83 deg/s, 1.67 deg/s, 3.33 deg/s and 6.67 deg/s), for motion in the same direction of movement as the target (‘with’) and for motion in the opposite direction to the target (‘against’). Twenty naïve participants completed this experiment with fifteen included in the analysis.

In experiment 1b we tested four speeds of higher magnitude (10 deg/s, 13.33 deg/s, 16.67 deg/s and 20 deg/s) for each of the two directions. Twenty-two participants completed this experiment, fifteen were included in the analysis, with three of these having also completed Experiment 1a.

### Results

**Fig 1:**
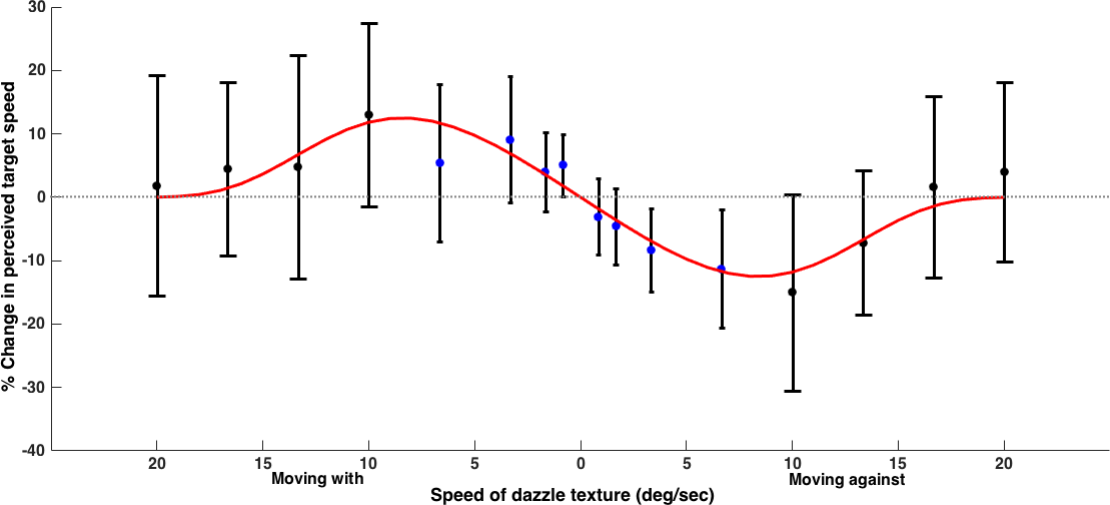
**Combined data for Experiments 1a (blue data points) and 1b (black data points).** Data points are means and error bars represent standard deviations (N = 15 for each experiment). The target speed was 10 deg/s. The effect on the perceived speed of the target peaks at 10 deg/s. ‘Speed of dazzle texture’ is defined as relative to the target not the background.

The combined data from both experiments are shown in Fig 1. There was a clear difference in perceived speed for the two directions of texture motion. When the texture moved in the same direction (‘with’) as the target rectangle, the perceived target speeds were faster than that of the static control. The opposite effect was shown when the texture moved against the direction of the target, with the perceived target speeds being slower.

The speed of the texture affected the change in perceived target speed: faster texture speeds increased the change in perceived target speed, up to a peak at 10 deg/s; beyond this value change in perceived target speed decreased as texture speed increased. This pattern occurred for both directions of the dynamic pattern and the relationship was modelled with a function of the form $y=-\frac{x}{a}\times sinc{\left(\frac{x^{2}-b}{c}\right)}^{4}$ using the fit function from the curve fitting toolbox in Matlab (Fig 1). The sinc function, sin(x)/x, was chosen because of its use in signal processing to represent a damped sine wave. We make no claims about the specific form of the relationship; the additional parameters were included simply to provide a good description of the data. The parameters and fit of the resulting model are shown in Table 1.

**Table 1:** Parameters and fit for the modelled relationship between speed of dazzle texture and change in perceived speed for the 10 deg/s target (Experiment 1) and the 5 deg/s target (Experiment 2).

Target speed	a	b	c	Adjusted r^2^
10 deg/s	3.966	-82.5	548.3	0.85
5 deg/s	0.002	-1772	2088	0.79

## Experiment 2: Generalizability of Speed Effects

In Experiment 1 it was shown that for a target speed of 10 deg/s the effect of the dynamic texture was maximised when the pattern also moved at 10 deg/s. Experiment 2 tested where the effect of the dynamic texture would peak when the target moved at a different speed: 5 deg/s.

### Methods

All experimental details were identical to Experiment 1 except that the target moved at 5 deg/s and the texture speed was adjusted accordingly: five different texture speeds were tested (1.25, 2.5, 5, 10 and 20 deg/s), in both ‘with’ and ‘against’ manipulations. The data were collected over two test sessions completed on consecutive days, with a static texture control in each session. This brought the total number of blocks to 12. In each session only one direction was tested and the order was counterbalanced across participants.

Twenty-one participants completed the study with fifteen included in the analysis, ten were completely new to the paradigm and five had previously participated in a pilot, experiment or both.

### Results

**Fig 2:**
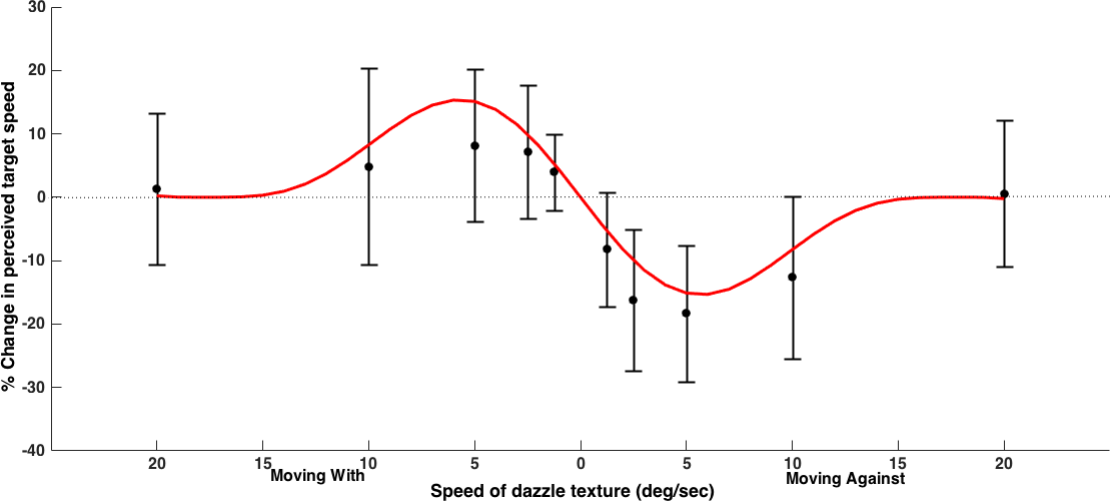
Data for Experiment 2 where the target speed was 5 deg/s. Points are means and error bars represent standard deviations (N = 15). As in Experiment 1, the effect on perceived speed was greatest when the texture speed matched the target speed.

As in Experiment 1, the speed distorting effect of the dynamic texture changed with texture speed, rising to a peak and then declining again. The effect was at its greatest when the texture speed matched the target speed of 5 deg/s. This was true of the both the ‘with’ and ‘against’ conditions, and the results were modelled with the same function as in Experiment 1 (Fig 2 and see Table 1 for parameters).

A comparison of the static control blocks provides no evidence for a difference in participant performance between sessions completed on consecutive days (paired samples *t*-test: *t*(14) = -0.85, *p* = .407).

## Experiment 3: Size and Position of Dynamic Camouflage

The evidence from Experiments 1 and 2 has shown the effect of dynamic texture to be both strong and predictable. Another important consideration for practical application is the logistics of having a moving texture on the side of a vehicle. Whilst the entire vehicle could be covered, the presence of doors that must be able to open means that it would be simpler if only certain sections of the vehicle required dynamic dazzle patterns. Is complete coverage necessary for effective speed distortion? We next investigated the effects of 50% and 25% coverage of the rectangular target in dynamic texture, placed at either the leading or trailing edge of the target. The remainder of the target was covered in a static stripe pattern with identical structure to the dynamic texture.

The importance of leading and trailing edges has been investigated previously. Arnold *et al*. [15] report not only that a static window containing motion can induce illusory positional shifts, but also that low-contrast targets positioned near such stimuli are easier to detect at the leading rather than trailing edge of motion. Whitney & Cavanagh [14] also employed stimuli consisting of a stationary window containing a moving grating; in a series of experiments they showed that peak fMRI activity occurred closer to the trailing edge of the motion. Through a combination of imaging and psychophysical results they concluded that the visual system contains a mechanism that specifically operates on the trailing edges of moving stimuli to suppress visual responses, possibly to diminish blur.

In the current experiment there are two sources of motion, the target itself and the dynamic texture that covers it. This therefore provides the opportunity not only to differentiate between the importance of the leading and trailing edges of the target, but also to investigate the importance of a correspondence between the leading and trailing edges of the dynamic texture and the target.

### Methods

**Fig 3:**
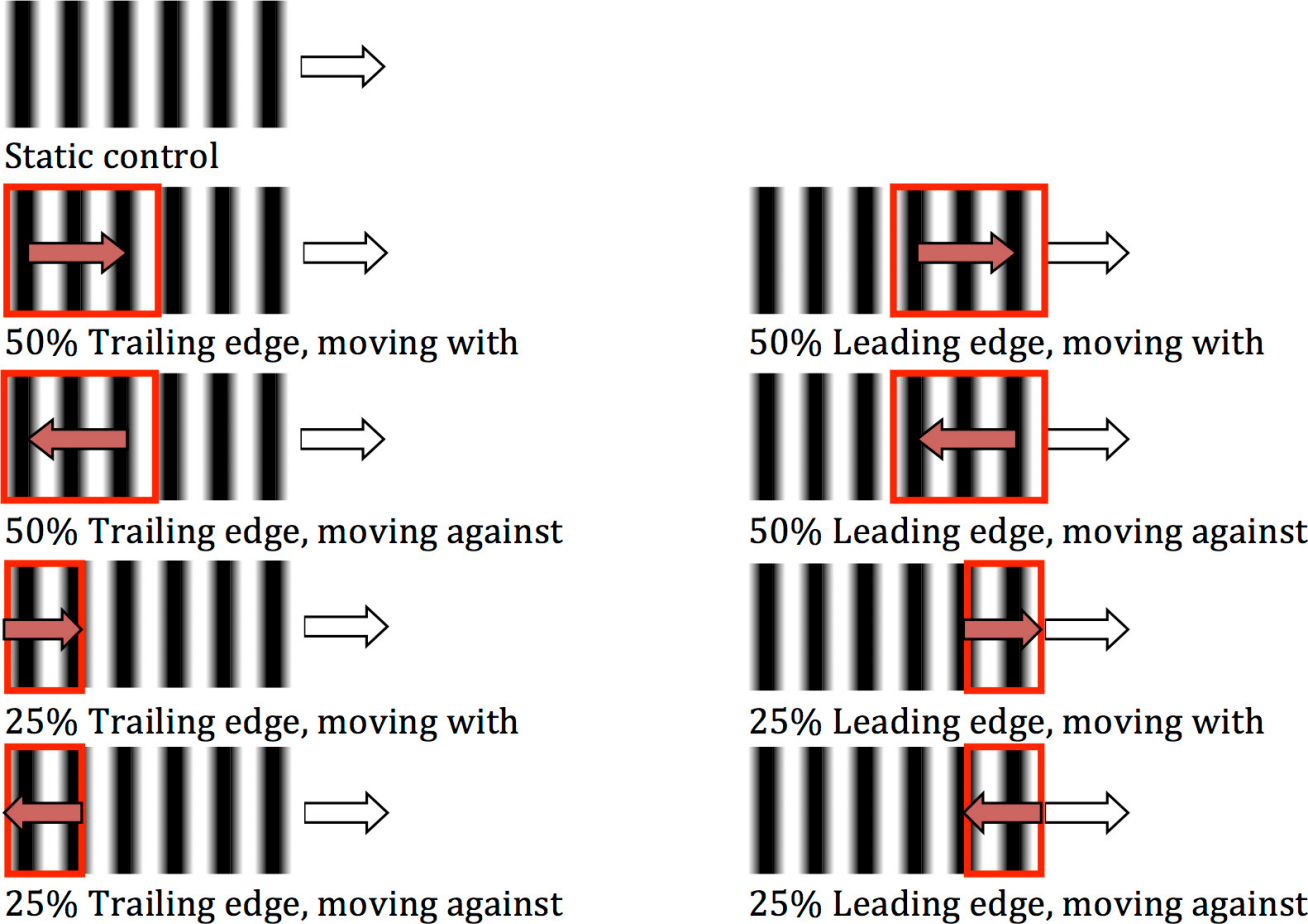
Conditions for Experiment 3. White arrow indicates direction of target and red arrow indicates direction of dynamic texture motion. Red rectangle indicates the proportion of the texture that is moving. The remainder of the texture is static.

The methods were as in the previous experiments, with the following exceptions. Size, position and direction of movement were manipulated. The patch of dynamic texture was either 25% or 50% of the area of the target rectangle and was positioned at either the leading or trailing edge, producing four combinations. The remainder of the target, not covered by dynamic texture, had a stationary pattern using the same grating as was used for the dynamic texture. The four combinations were tested for motion ‘with’ and ‘against’, producing a total of eight blocks of test stimuli (Fig 3). Again there was a ninth block containing the static control. The texture speed was constant at 3.3 deg/s and the target speed was 10 deg/s. This texture speed was selected to allow the possibility of either an increase or a decrease in the speed distortion from a baseline of around 10% (as determined in Experiment 1). Twenty-one participants completed this experiment with fifteen included in the analysis, of which five had also completed a previous experiment or pilot.

### Results

Out of the eight conditions tested only two produced a significant change in the perceived speed of the target compared to the static texture control (see Table 2). The significant changes were seen in the two conditions where the dynamic camouflage was positioned at the trailing edge and moved in the same direction as the target. The mean increase in perceived speed was around 6% for the 25% coverage patch, and 9% for the 50% coverage patch; however there was no significant difference between them (paired samples *t*-test, *t*(14) = 1.60, *p =* .132), suggesting that the size of the patch had little impact on the perceived speed of the target.

**Table 2:** Experiment 3: Mean (±SD) change in perceived speed, compared to static dazzle control, and one-sample *t*-test results for different combinations of dazzle size and position.

Direction of dazzle	Position of dazzle	Proportion of target covered by dazzle	Mean change in perceived speed (deg/sec)	*t*	df	*p*
With	Leading edge	0.25	0.30 (1.07)	1.07	14	*p* = .305
With	Leading edge	0.5	0.48 (1.21)	1.56	14	*p* = .142
With	Trailing edge	0.25	0.62 (1.07)	2.23	14	*p* = .042
With	Trailing edge	0.5	0.93 (1.09)	3.31	14	*p* = .005
Against	Leading edge	0.25	0.02 (1.28)	0.06	14	*p* = .952
Against	Leading edge	0.5	-0.21 (0.83)	-0.95	14	*p* = .358
Against	Trailing edge	0.25	0.01 (0.97)	0.51	14	*p* = .960
Against	Trailing edge	0.5	-0.07 (0.79)	-0.36	14	*p* = .727

Statistics from one-sample *t*-tests (compared to 0)

## Experiment 4: Double versus Single Patches

As single edges proved largely ineffective, Experiment 4 compared the effect of a single patch of dynamic texture at the trailing edge of the target (the only successful manipulation in Experiment 3) with two patches (of equal total area) of dynamic texture split between both ends. So, for example, a single patch of 50% of the area of the target was compared to two patches of 25% of the area of the target.

While the visual system may preferentially attend to motion signals from the trailing edge of moving objects, further information about the motion of the object could also be gained from the leading edge. If the visual system combines the information from both edges, then having dynamic texture patches at each end of the target might increase the change in perceived speed when compared to a single patch at the trailing edge.

If, as hypothesised above, it is important for the trailing edges of the target and the dynamic texture patch to match up, the change in perceived speed due to patches at both ends of the target should be greater when the texture patches are moving ‘with’ the target than when they are moving ‘against’.

### Methods

**Fig 4:**
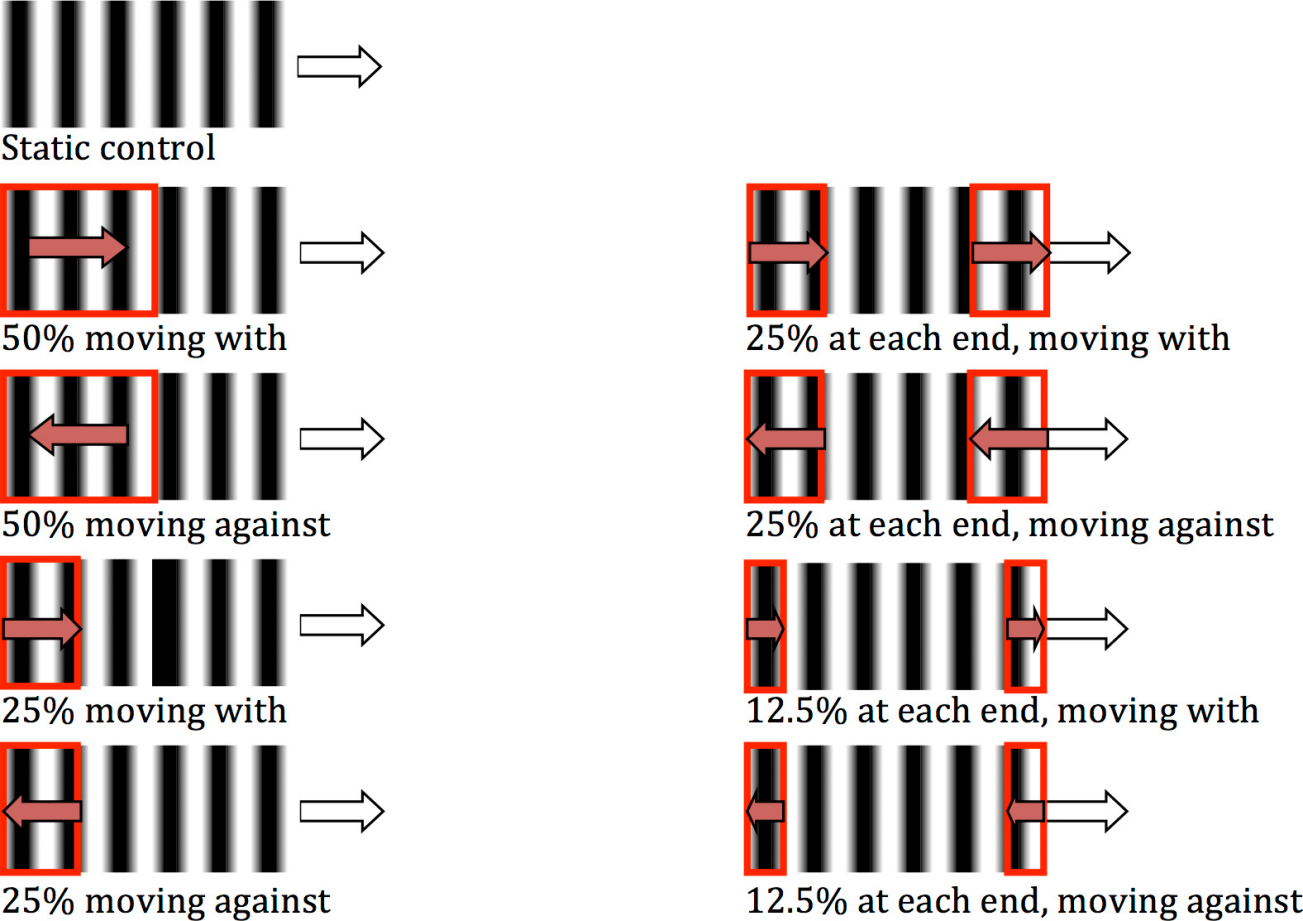
Conditions for Experiment 4. White arrow indicates direction of target and red arrow indicates direction of dynamic texture. Red rectangle indicates proportion of dynamic texture. The remainder of the texture is static.

The methods were as previously (target speed of 10 deg/s and texture speed of 3.33 deg/s) with a static control condition and four conditions for each direction: 50% area at trailing edge, 25% area at each edge, 25% area at trailing edge and 12.5% area at each edge (see Fig 4). Eighteen participants completed this experiment with fifteen included in the analysis, of which five had completed a previous experiment or pilot.

### Results

**Fig 5:**
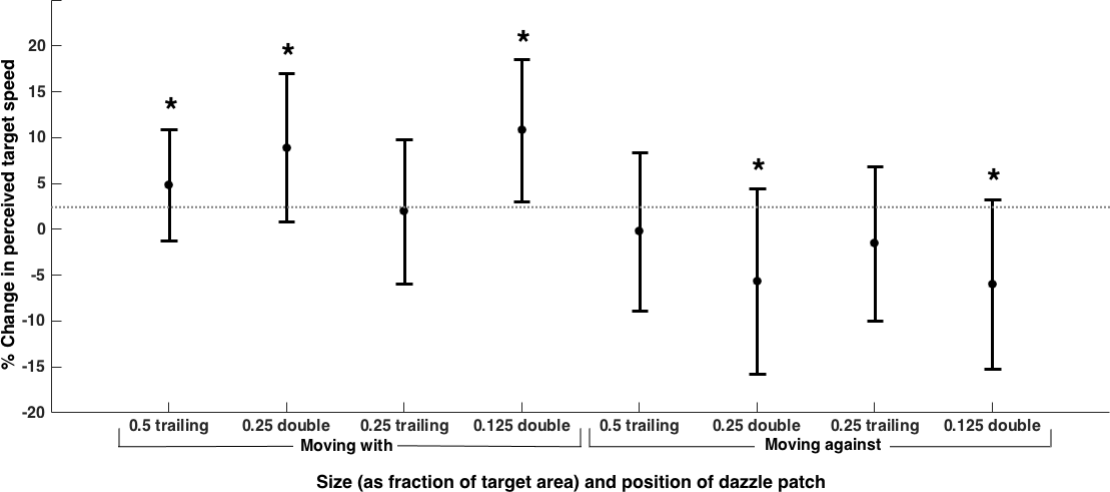
Mean (±SD) percentage change in perceived speed for Experiment 4 (N = 15 participants). Dynamic texture was present only at the trailing edge of the target or at both ends (‘double’). *Indicates change in perceived speed is significantly different from zero. The effect on perceived speed was greatest for conditions where there was a patch of dynamic texture at each end (‘double’), even when the texture at the trailing edge covered an identical proportion of the target.

In all cases splitting the dynamic texture patch across both edges led to a significant change in perceived speed compared to the control (see Fig 5), while 50% coverage at the trailing edge only led to a significant change for the ‘with’ direction and 25% coverage at the trailing edge had no detectable effect on the perceived speed of the target (see Table 3 for results of one sample *t*-tests)

**Table 3:** One-sample *t*-test results for different combinations of dazzle size and number of patches.

Direction of dazzle	Position of dazzle patches	Proportion of target covered by each patch	*t*	df	*p*
With	Trailing	0.5	3.13	14	*p* = .007
With	Double	0.25	4.330	14	*p* = .001
With	Trailing	0.25	0.97	14	*p* = .350
With	Double	0.125	5.41	14	*p* < .001
Against	Trailing	0.5	-1.01	14	*p* = .328
Against	Double	0.25	-2.15	14	*p* = .049
Against	Trailing	0.25	-0.70	14	*p* = .498
Against	Double	0.125	-2.52	14	*p* = .025

Statistics from one-sample *t*-tests (compared to 0)

A 2x2x2 repeated measures ANOVA with the magnitude of the change in perceived speed as the dependent variable, showed that the equivalent surface area of patch located across both edges was significantly more effective than when it was located at only the trailing edge. The amount of surface area that was covered by the moving dazzle patches had little effect when there was a patch at each edge of the target. The smallest surface area split between the two ends (12.5% at each end) was as effective as having twice the surface area at each end (25%). There was also no difference in the magnitude of the change between the two directions (main effect of patch position: F(1,14) = 8.70, *p* = .011; main effect of direction: F(1,14) = 0.001, *p* = .976; main effect of surface area: F(1,14) = 0.339, *p* = .570; patch position*direction: F(1,14) = 2.16, *p* = .164; patch position*surface area: F(1,14) = 1.04, *p* = .325; direction*surface area: F(1,14) = 0.55, *p* = .470; patch position*direction*surface area: F(1,14) = 0.11, *p* = .746).

## Experiment 5: Contrast of Dynamic Camouflage

There is evidence that a reduction in contrast can change the perceived speed of a target. Thompson *et al*. [22, 23] reported that for slow moving targets a reduction in contrast reduces perceived speed, whilst for fast moving targets (>8Hz, 4deg/s) reducing contrast increases perceived speed. However, Scott-Samuel *et al*. [9] found no effect on perceived speed of any low contrast patterns at either low or high speed, despite an effect at high contrast for some patterns. It is, therefore, not clear whether contrast would have any impact on the change in perceived speed due to the dynamic texture. However, the dynamic texture and targets are relatively fast moving and so one possibility is that reducing the contrast will increase the perceived speed of the dynamic texture and thus increase the change in perceived speed of the target.

### Methods

In the previous experiments, the grating stimuli were displayed at 100% contrast; here contrast was systematically reduced. There were nine conditions, split into three different contrast levels: 100%, 25% and 6.25%. For each contrast level, with and against conditions were tested and compared to a static condition. Again texture speed was 3.33 deg/s and target speed was 10 deg/s. Twenty-six participants completed this experiment, fifteen were included in the analysis and none had completed any of the previous experiments or pilots in this series.

### Results

**Fig 6:**
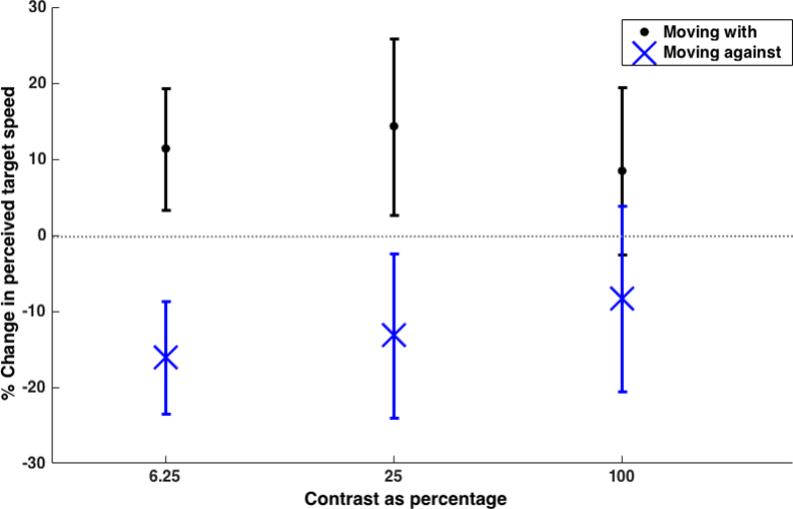
Mean (±SD) percentage change in perceived speed for Experiment 5 (N = 15 participants). All manipulations of the level of contrast in the texture produced a change in perceived speed that was significantly different from zero.

For all contrasts the perceived speed increased when the texture moved with the target and decreased when the texture moved in the opposite direction to the target (see Fig 6).

All of the dynamic patterns showed a statistically significant effect at changing the perceived speed of the target compared to the static control with relevant contrast (see Table 4) A repeated measures ANOVA, with magnitude of change in perceived speed as the dependent variable, direction as a fixed factor and contrast modelled as a continuous factor, showed that reducing the contrast of the grating did not reduce the effectiveness of the dynamic texture at changing the perceived speed of the target (main effect of direction: F(1,14) = 1.01, *p* = .333; main effect of contrast: F(1,14) = 1.75, *p* = .207; interaction: F(1,14) = 0.54, *p* = .475).

**Table 4:** One-sample *t*-test results for differences between dynamic dazzle and static dazzle control for each of the 3 contrast levels tested.

Direction of dazzle	Contrast (as %)	*t*	df	*p*
With	100	2.98	14	*p* = .010
Against	100	-2.66	14	*p* = .019
With	25	4.78	14	*p* < .001
Against	25	-4.69	14	*p* < .001
With	6.25	5.49	14	*p* < .001
Against	6.25	-8.45	14	*p* < .001

Statistics from one-sample *t*-tests (compared to 0)

## Experiment 6: Adding Colour to Dynamic Camouflage

Dazzle patterns are generally conceived as being black and white; however, the original dazzle patterns used on ships were not always monochrome [6,7]. In order for dynamic dazzle to have a real world application, in some contexts it would be desirable for colour to be introduced into the patterns. Black and white patterns are not an effective camouflage strategy in terrestrial environments where vehicles will sometimes need to be stationary and concealed, as well as moving and protected by dynamic dazzle at others. Black and white patterns would likely cause a stationary vehicle to stand out against its background and therefore make it an easy target. The use of colours that blend into the background when the vehicle is stationary, but which are still able to elicit a change in perceived speed when it is moving, would provide a useful solution to this problem. We therefore tested a dynamic texture with green and brown colouration.

### Methods

**Fig 7:**
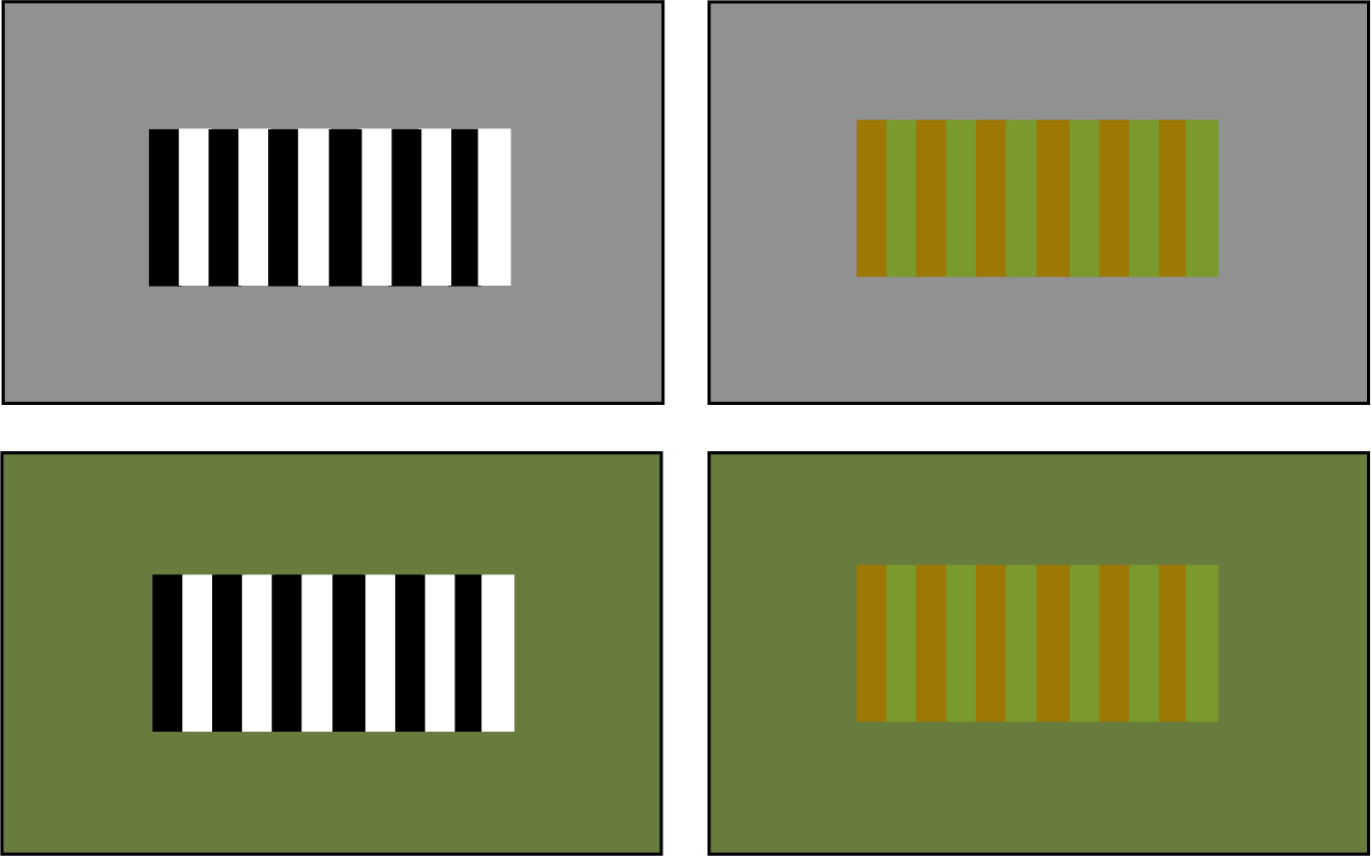
Colour combinations for Experiment 6. Each combination was tested for the moving ‘with’ and moving ‘against’ directions, producing eight conditions.

Stripes could be either monochrome or coloured. However, these were square waves as opposed to sinusoidal gratings because, again with an eye to practical applications, it is easier to paint solid blocks of colour than gradients. The background was either grey (mean luminance) or green (RGB values set on monitor: 85 107 47; CIE Yxy colour space values from photometer measurement: 65.8 0.277 0.422) and the stripes were either black and white or green (RGB: 105 139 34; CIE Yxy: 79.2 0.299 0.468) and brown (RGB: 139 101 8; CIE Yxy: 61.5 0.361 0.496). This produces four combinations of background and stripes (Fig 7), which were then tested for both directions of texture motion, giving eight conditions. In all cases texture speed was 3.33 deg/s and target speed was 10 deg/s. Twenty-four participants completed this experiment with fifteen included in the analysis, of which two had completed a previous experiment or pilot.

### Results

A 2x2x2 repeated measures ANOVA showed a main effect of direction (F(1,14) = 98.519, *p* < .001) with the perceived speed of the target being faster when the texture was moving in the same direction as the target (mean: 11.32 ± 1.17 deg/s) and slower when the texture was moving in the opposite direction to the target (mean: 9.25 ± 0.75deg/s). There was also a main effect of stripe colour (F(1,14) = 5.329, *p* = .037) with black and white stripes leading to a slight increase in perceived speed (mean: 10.40 ± 0.86 deg/s) compared to brown and green stripes (mean: 10.17 ± 0.95 deg/s). There was no effect of the background colour (F(1,14) = 3.013, *p* = .105).

**Fig 8:**
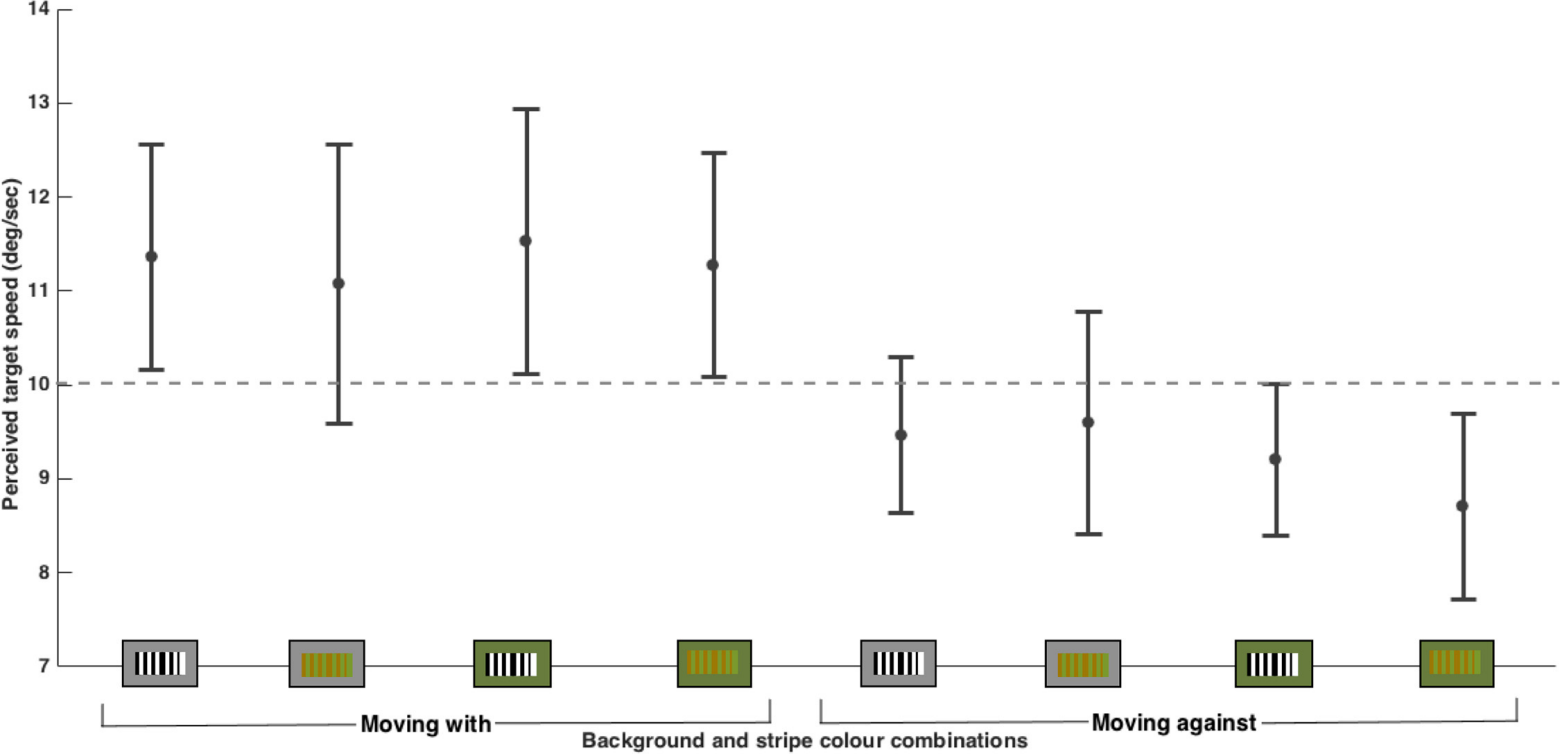
Mean perceived target speeds (± SD) for different colour combinations in Experiment 6 (N = 15 participants). The effect of the texture was enhanced by the green background so that the perceived speed was increased for the moving with direction, and decreased for the moving against direction, when compared to the grey background.

The interaction between direction and background was significant however, (F(1,14) = 11.458, *p* = .004) with the green background enhancing the effect of the texture on the perceived speed. In the moving ‘with’ direction the perceived speed was increased compared to that for the grey background and for the moving ‘against’ direction the perceived speed was decreased (see Fig 8). None of the other interactions were significant (direction*stripes: F(1,14) = .066, *p* = .800; background*stripes: F(1,14) = 4.189, *p* = .060; direction*background*stripes: F(1,14) = .913, *p* = .356).

## Experiment 7: Effects of Stress on Perceived Speed Distortions

In military applications of a dynamic dazzle strategy, the intended observer would likely be experiencing greater levels of stress and anxiety than the undergraduate experimental participants on whom the strategy has so far been tested. It is well established in the anxiety literature that anxious individuals display an attentional bias to threat related stimuli (for a review see [24]). Such an attentional bias could potentially be directed towards the intended target in individuals experiencing anxiety due to their intended actions. It is therefore important to establish whether the dynamic texture is able to change the perceived speed of the target as successfully under stressful conditions as it has so far been shown to under neutral conditions.

The 7.5% carbon dioxide challenge is a technique that provides an experimental model of generalised anxiety in healthy humans [25]. Inhalation of air containing higher-than-normal concentrations of carbon dioxide increases both self-report anxiety and autonomic arousal, such as heart rate and blood pressure. Previous studies using the 7.5% carbon dioxide challenge have shown that along with increased anxiety, CO_2_ inhalation increased eye movements towards threat stimuli [26] and increased attention to the temporal and spatial location of stimuli [27]. The latter could potentially disrupt any effects of dynamic dazzle patterns as greater attention to the location of the target may reduce any illusory speed effects.

In order to test whether the effect of the dynamic texture is robust to stressful conditions, participants were asked to complete a dynamic texture task whilst undergoing the 7.5% CO_2_ challenge.

### Methods

#### CO_2_ Challenge

Seventeen participants completed the experiment of which fifteen were included in the analysis. None of the participants had previously experienced the CO_2_ challenge, and 14 of those included in the analysis were also new to the dynamic texture paradigm with one having participated in previous experiments. Participants were aged between 18 and 40 years, in good physical and psychiatric health (assessed by a short structured interview based on the Mini-International Neuropsychiatric Interview; MINI) with no recent use of prescribed medication (within 8 weeks of study session). Other exclusion criteria included daily smoking, pregnancy (verified by urine screen) or breast-feeding, asthma, history of migraine and recent use of illicit drugs (verified by urine screen). Diastolic blood pressure (DBP: < 90 mm Hg), systolic blood pressure (SBP: < 140 mm Hg), heart rate (50–90 bpm) and body mass index (BMI; 18–30 kg/m2) were required to be within the normal range. Prior to the study, participants were asked to refrain from alcohol for 24 h and (if an irregular smoker) to refrain from smoking for 12 h. Participants were reimbursed £25 for their time at the end of testing. The study was conducted in line with the Declaration of Helsinki, and was reviewed and approved by the University of Bristol Faculty of Science Research Ethics Committee. All participants gave full written informed consent.

Once all screening was complete, initial measures of, heart rate, blood pressure, subjective state anxiety [28], and positive/negative affect [29] were recorded for use as a baseline. These measures were then repeated immediately after each of the inhalations. Participants were asked to answer the questionnaires based on how they felt when the effects of the inhalation were at their greatest.

Participants performed the dynamic texture task during two inhalations, one of 7.5% CO_2_ enriched air (21% O_2_, balance N_2_) and one of medical (normal) air. Each inhalation lasted a maximum of 20 min and there was a 30 min break between inhalations. The order of the inhalations was counterbalanced across participants. The gas was administered through an oro-nasal facemask and participants were not aware of the order in which they would experience the inhalations (for further details of procedures for the CO_2_ challenge see Materials and Methods section in [30]).

#### Dazzle task

The dynamic texture patterns consisted of gratings, with 100% contrast and 100% coverage of the target. As previously, the target moved at 10 deg/s and the texture speed was 3.33deg/s. Since the inhalations were limited to 20 min, it was only possible to present 3 conditions during each, so the texture was presented moving in both the ‘with’ and ‘against’ directions, along with a static control.

### Results

#### Effects of 7.5% CO_2_ inhalation on physiology and mood

Inhalation of 7.5% CO_2_ induced feelings of stress and anxiety as measured by changes in both physiology and mood. There were significant increases in anxiety, negative affect and heart rate and a significant decrease in positive affect for the CO_2_ inhalation compared to the normal air inhalation (see Table 5). There was no difference in blood pressure between the two inhalations.

**Table 5:** Mean (±SD) for anxiety, mood, blood pressure and heart rate measures following inhalations of 7.5% CO_2_ and normal air.

	Air	7.5% CO_2_	*t*	df	*p*
State anxiety	30.9 (7.0)	55.0 (11.9)	8.68	14	*p* < .001
Negative affect	11.0 (2.0)	19.6 (6.0)	6.36	14	*p* < .001
Positive affect	27.1 (5.2)	18.7 (6.9)	-5.16	14	*p* < .001
Systolic blood pressure	115.1 (13.2)	120.0 (14.6)	1.34	14	*p* = .203
Diastolic blood pressure	69.3 (15.1)	64.9 (5.7)	-1.10	14	*p* = .289
Heart rate	65.9 (8.4)	81.6 (16.9)	4.62	14	*p* < .001

Statistics are from paired *t*-tests.

#### Effects of adaptive camouflage on perceived speed in neutral situations

**Fig 9:**
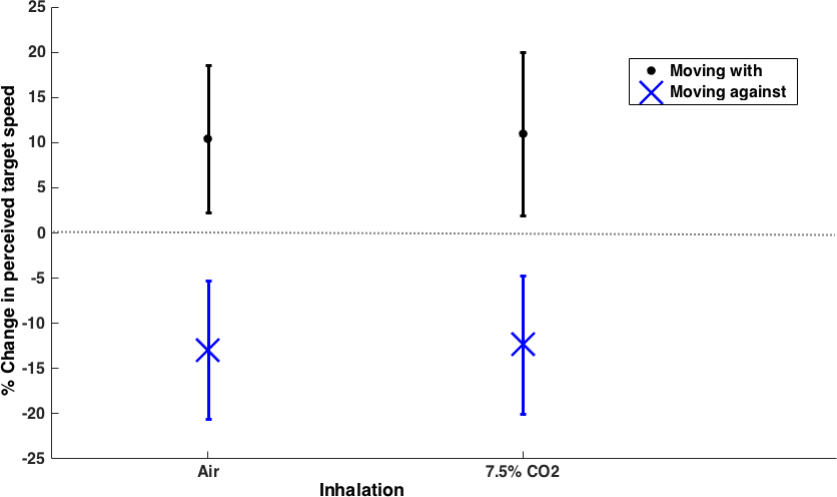
Mean (±SD) percentage change in perceived speed for the two inhalations conditions (N = 15 participants). The dynamic texture causes the target to be perceived as moving faster or slower depending on the direction of the camouflage movement. This appears to be robust to stressful situations as the effect is similar for both normal air and 7.5% CO_2_ inhalations.

With the inhalation of normal air, the dynamic texture produced the expected effect where perceived speed increases when the pattern moves with the target and decreases when the pattern moves in the opposite direction (Fig 9).

There was a significant increase, of about 10%, in the magnitude of the change in perceived speed for both directions of texture movement compared to the static control (moving ‘with’ change: mean = 1.04 ± 0.25 deg/s, one-sample *t*-test, *t*(14) = 4.95, *p* = .0002; moving ‘against’ change: mean = -1.29 ± 0.24 deg/s, one-sample *t*-test, *t*(14) = -6.55, *p* < .0001). This is consistent with the results from Experiment 1.

#### Effects of adaptive camouflage on perceived speed in stressful situations

While the 7.5% CO2 inhalation increased the stress and anxiety experienced by the participants compared to the normal air inhalation, there was no difference between the perceived speeds of the control stimuli for the two inhalations (air mean = 3.06 ± 0.27; 7.5% CO_2_ mean = 2.99 ± 0.24; paired samples *t*-test, *t*(14) = 1.22, *p* = .24).

For both the air and CO_2_ inhalations there were significant increases, of about 10%, in the magnitude of the change in perceived speed for both directions of camouflage movement compared to the static control (moving ‘with’ change: mean = 1.10 ± 0.28 deg/s, one-sample *t*-test, *t*(14) = 4.69, *p* = .0003; moving ‘against’ change: mean = -1.23 ± 0.24 deg/s, one-sample *t*-test, *t*(14) = -6.22, *p* < .0001, see Fig 9). The magnitude of the change in perceived speed, compared to the static control, was tested in a 2x2 repeated measures ANOVA to establish whether there was a difference in the size of the effect for the two types of inhalation. There was no significant effect of inhalation or target direction and there was no evidence for an interaction between these factors (inhalation: F(1,14) = .176, *p* = .681; direction: F(1,14) = .312, *p* = .585; interaction: F(1,14) = .210, *p* = .654). The effects of the dynamic texture were therefore similar for the 7.5% CO_2_ inhalation and the normal air inhalation.

## General Discussion

Previous work has provided evidence that some static, high contrast patterns are able to affect the perceived speed of an object [4, 5, 9] and that moving gratings inside a window can induce illusory changes in location [12–15] and speed [16]. In this series of experiments we combined these two strategies to create and test a dynamic texture: a high contrast, moving pattern on a moving target. We have shown that this dynamic texture can successfully change the perceived speed of a target, either increasing or decreasing the speed, depending on the direction of the pattern relative to the direction of the target motion.

The effect on the perceived speed is much larger than that found previously for static patterns and can be as great as 18% for certain conditions. Scott-Samuel *et al*. [9] found that stationary, two dimensional, zigzag patterns could slow the perceived speed of a target moving at 20 deg/s by about 7%. In these current experiments we have shown that a moving, one-dimensional grating can both reduce and increase the perceived speed of a target, depending on the direction of motion of the pattern. At its maximum effect, the dynamic texture was able to change the perceived speed by double that of stationary zigzags, for a target that was moving at half the speed of the targets used by Scott-Samuel *et al*.

The pattern of results produced for the two target speeds that were tested in Experiments 1 and 2 were very similar, displaying a curve that peaked at the point where the speeds of the texture and target matched. This pattern suggests that the effects of the dynamic texture could be easily predicted for other target speeds. This is a particularly useful property for real world applications as it means the target speed could easily be manipulated and varied, whilst still producing predictable effects.

Scott-Samuel *et al*. [9] reported that for static dazzle patterns, only two-dimensional patterns, such as zigzags, changed the perceived speed of a moving target. However, with the introduction of motion to the texture pattern, one-dimensional patterns can change the perceived speed of the target. A square wave (*i*.*e*. hard-edged stripes) was found to be as effective as a grating (*i*.*e*. soft-edged stripes) at modulating the perceived speed and, while the full range of dynamic texture speeds was not tested for this pattern, its similarity in appearance to a grating makes it highly likely that it would perform in a similarly predictable manner. This point could easily be verified in the future. One big advantage of using stripes as opposed to a grating is the ease of application for use in the real world. Stripes are much easier to produce both in black in white and, most importantly, for different colour combinations.

Manipulations of the size and position of the texture patch showed that the visual system preferentially attends to the trailing edges of moving objects. Consistent with Whitney & Cavanagh [14], the trailing edge of the target was shown to be more important than the leading edge for speed perception; placing a single dynamic texture patch at the leading edge had no detectable effect while a single dazzle patch at the trailing edge was able to change the perceived speed of the target. The dynamic texture patch at the trailing edge was only effective at changing the perceived speed of the target when it was moving in the same direction as the target. A possible explanation for this result is that when the dynamic texture is at the trailing edge of the target but moving in the opposite direction, the trailing edge of the target and the trailing edge of the dynamic texture motion do not coincide. However, when the dynamic texture moves ‘with’ the motion of the target the trailing edge of each falls in the same place. We conclude that a correspondence between the trailing edges of the target and the dynamic texture is an important factor when implementing this strategy to change perceived speeds. Overall, the data from this manipulation suggest that speed distortion effects with single edges of dynamic texture are modest.

Whilst the dynamic texture was found to be most effective when covering the entire target, the presence of a small patch at either end of the target was also shown to be successful. Regardless of the total surface area covered by the patches, in all cases tested, placing a patch of dynamic texture at both the leading and trailing edges of the target increased the change in perceived speed, compared to the equivalent surface area displayed as a single patch at the trailing edge. It therefore appears that while the visual system does preferentially attend to the trailing edge of a moving object, it is able to integrate information from both the leading and trailing edges when judging speed. There was a small reduction in the effect on perceived speed resulting from the use of two smaller patches rather than a single patch that covered the entire target, probably due to the reduced strength of the signal. The fact that there was still a change in perceived speed indicates that the visual system allocates limited attention to the central section of a moving object, using it simply to reinforce the signal perceived from the trailing, and to some extent leading, edges. In real world applications, the slight reduction in effect on perceived speed might be offset by the reduced cost of only having to display a moving pattern at each end of a vehicle, rather than covering the entire side, potentially including doors that need to open.

Manipulations of the contrast of the texture patch showed that the magnitude of the change in perceived speed induced by the dynamic texture was the same for all the contrast levels tested. This bodes well for real world application of the dynamic texture strategy. In addition, the introduction of colour, in this case green and brown, did not abolish the effects of the dynamic texture and the use of a green background increased the magnitude of the change in perceived speed for both directions of movement, compared to a grey background. This is particularly important for real world applications where the camouflage would need to provide protection when a target was static (through background matching), as well when it moved (through speed distortion). For background matching to be effective, the colours would need to mimic those that are common in the environment and so it would be advantageous if the requirements for the speed distortion effects were not to restrict the colour choices available for the camouflage. Stevens, Yule & Ruxton [5] tested uniform and high contrast conspicuous patterns, and reported that targets were more difficult to capture when displayed against more heterogeneous backgrounds. This bodes well for this dynamic texture strategy as it has so far been tested against uniform backgrounds and yet still produced a clear effect. Further tests would be required in order to characterise the behaviour of the illusion with a full range of colour combinations.

Despite the fact that anxiety can lead to attentional biases that increase attention to temporal and spatial locations of stimuli [27], the dynamic texture strategy appears to be robust to the effects of anxiety. Increases in heart rate and self-report anxiety indicate that the participants did experience anxiety and stress during the inhalation of CO_2_ enriched air; however, there was no difference in the effects of the dynamic texture on the perceived speed of the target between the CO_2_ and normal air conditions. The stressful conditions neither increased nor decreased the effects of the dynamic texture. This is an important result for the potential use of the dynamic texture for real world applications and means that the effect remains predictable even during stressful conditions.

Changes in perceived speed of around 15% were seen when the target moved at 5 or 10 deg/s and was entirely covered by the dynamic texture. For a Land Rover at 70m, this corresponds approximately to a targeting error of 0.5m if the vehicle is travelling at 13mph and 1m if it is travelling at 25mph. Assuming the magnitude of the effect would be similar for a target moving at 20 deg/sec, this would correspond to a targeting error of 2m for a Land Rover travelling at 55mph, double that found by Scott-Samuel et al [9] for static dazzle patterns. Whether the Land Rover was perceived to be moving faster or slower than its actual speed, this targeting error would be enough to reduce the risk of vehicle occupants receiving a direct hit. The ability to manipulate the illusion in both directions also increases the unpredictability of the signal as perceived by any observers. Further considerations would be required when scaling the illusion for use on an object of a similar size to a Land Rover. The distance from which the target is likely to be viewed is important as the stripes will start to blend as viewing distance increases. This could prove to be advantageous if it increased the background matching of the target when viewed from a distance outside the range of a handheld weapon. If the viewing distance at which the illusion fails was too close to the target, the spatial frequency of the pattern could be manipulated to increase the width of the stripes; however, this would need to be balanced with the fact that there will be a minimum proportion of the pattern that must be seen for the illusion to work effectively. Another consideration would be the texture speed required if the vehicle was travelling at high speed. After a certain threshold the speed of the texture would cause the stripes to blend together or start to flicker which would destroy the illusion. Although the effect of the illusion was found to be maximised when the texture speed matched the target speed, this was not required for there to be a change in perceived speed. One potential solution would be for the texture to move at a slower speed than the target, thus still providing protection and removing the risk of flickering.

The ability of the dynamic texture to change the perceived speed of a target suggests that it should be considered a form of dynamic dazzle camouflage. Whilst the potential military applications may be obvious, the characterisation of this illusion not only furthers our understanding of the human visual system, but may also be relevant to understanding the function of dynamic displays in animals such as cephalopods [31] and patterns of iridescence in insects. Iridescence is a change in observed hue due to a change in visual angle. Multilayer iridescence is common among a wide variety of insects including beetles and butterflies (e.g.[32,33]) and the exact structures in the layers affect the wavelength of light that is reflected. In theory, if the structures were laid down in a manner to produce a repeating pattern (e.g. stripes) then movement of the insect or observer would change the visual angle, causing the hues to change, and producing a signal similar to dynamic dazzle.

Overall, dynamic dazzle based on a one dimensional pattern appears to be a predictable and robust strategy for changing the perceived speed of a target, with evidence suggesting that the effect is also immune to stress.
